# A Machine Learning-Based Decision Support System for the Prognostication of Neurological Outcomes in Successfully Resuscitated Out-of-Hospital Cardiac Arrest Patients

**DOI:** 10.3390/jcm13247600

**Published:** 2024-12-13

**Authors:** Sijin Lee, Kwang-Sig Lee, Sang-Hyun Park, Sung Woo Lee, Su Jin Kim

**Affiliations:** 1 Department of Emergency Medicine, Korea University Anam Hospital, Seoul 02841, Republic of Korea; reonoaz85@gmail.com (S.L.); kuedlee@korea.ac.kr (S.W.L.); 2 AI Center, Korea University College of Medicine, Seoul 02841, Republic of Korea; ecophy@korea.ac.kr; 3 Biomedical Research Institute, Korea University College of Medicine, Seoul 02841, Republic of Korea; lovephysics@korea.ac.kr

**Keywords:** out-of-hospital cardiac arrest, neurological outcome, machine learning, random forest, decision support system

## Abstract

**Background/Objectives:** This study uses machine learning and multicenter registry data for analyzing the determinants of a favorable neurological outcome in patients with out-of-hospital cardiac arrest (OHCA) and developing decision support systems for various subgroups. **Methods:** The data came from the Korean Cardiac Arrest Research Consortium registry, with 2679 patients who underwent OHCA aged 18 or above with the return of spontaneous circulation (ROSC). The dependent variable was a favorable neurological outcome (Cerebral Performance Category score 1–2), and 68 independent variables were included, e.g., first monitored rhythm, in-hospital cardiopulmonary resuscitation (CPR) duration and post-ROSC pH. A random forest was used for identifying the major determinants of the favorable neurological outcome and developing decision support systems for the various subgroups stratified by the major variables. **Results:** Based on the random forest variable importance, the major determinants of the OHCA patient outcomes were the in-hospital CPR duration (0.0824), in-hospital electrocardiogram on emergency room arrival (0.0692), post-ROSC pH (0.0579), prehospital ROSC before emergency room arrival (0.0565), coronary angiography (0.0527), age (0.0415), first monitored rhythm (EMS) (0.0402), first monitored rhythm (community) (0.0401), early coronary angiography within 24 h (0.0304) and time from scene arrival to CPR stop (0.0301). It was also found that the patients could be divided into six subgroups in terms of their prehospital ROSC and first monitored rhythm (EMS), and that a decision tree could be developed as a decision support system for each subgroup to find the effective cut-off points regarding the in-hospital CPR duration, post-ROSC pH, age and hemoglobin. **Conclusions:** We identified the major determinants of favorable neurological outcomes in successfully resuscitated patients who underwent OHCA using machine learning. This study demonstrates the strengths of a random forest as an effective decision support system for each stratified subgroup (prehospital ROSC and first monitored rhythm by EMS) to find its own optimal cut-off points for the major in-hospital variables (in-hospital CPR duration, post-ROSC pH, age and hemoglobin).

## 1. Introduction

Out-of-hospital cardiac arrest (OHCA) is a major health problem with a great regional variation in its incidence and neurological outcome [1]. Its global rate of survival to discharge is estimated to be 8.8%, with a wide regional variation, from 4.5% in Asia and 7.7% in North America to 11.7% in Europe and 16.2% in Oceania [2]. Likewise, its regional rates of a good neurological outcome show a great difference, within a range of 2–8% [3,4,5]. Specifically, in South Korea, as many as 30,000 OHCA cases are reported every year, but its rates of survival to discharge and a good neurological outcome are as low as 8.7% and 5.1%, respectively [6]. Two reviews [2,7] and several studies [5,7,8,9,10] have highlighted the following variables as the key predictors of a good neurological outcome: younger age, male status, the event being witnessed by a bystander or emergency medical services (EMS) provider, the provision of bystander cardiopulmonary resuscitation (CPR), the first monitored rhythm of ventricular fibrillation (VF) or pulseless ventricular tachycardia (pVT) and the prehospital return of spontaneous circulation (ROSC). These are popular predictors considered in traditional linear/logistic regressions. However, these predictors are “prehospital” predictors determined out of the hospital; hence, the existing literature centering on these prehospital predictors presents limited information on effective interventions for patients who underwent OHCA in an emergency department (ED). It should be noted that recent machine learning studies suffer from these limitations as well. These machine learning studies of predicting the outcomes of OHCA patients have shown their superior performance to linear/logistic regressions [11,12,13,14], but they lack sufficient in-hospital information for evaluating the risk–benefit of invasive interventions or deciding on the withdrawal of life-sustaining treatment.

This study was designed to (1) discern in-hospital variables and other key predictors of favorable neurological outcomes in patients who underwent OHCA and then (2) build decision support systems for the outcomes considering the in-hospital factors of the various subgroups stratified by the major key factors.

This study employs machine learning to accomplish this aim and goes beyond the scope of traditional research in two aspects. Firstly, this study pays due attention to the prehospital and in-hospital variables for the prediction of outcomes. Secondly, this study uses machine learning and multicenter registry data to form various subgroups stratified by the major predictors as the first stage and develops a decision support system for each subgroup as the second stage. Specifically, this study highlights the strengths of a random forest as an effective decision support system for each subgroup to find its own optimal cut-off points for important continuous clinical variables.

## 2. Methods

### 2.1. Participants and Variables

**Figure 1:**
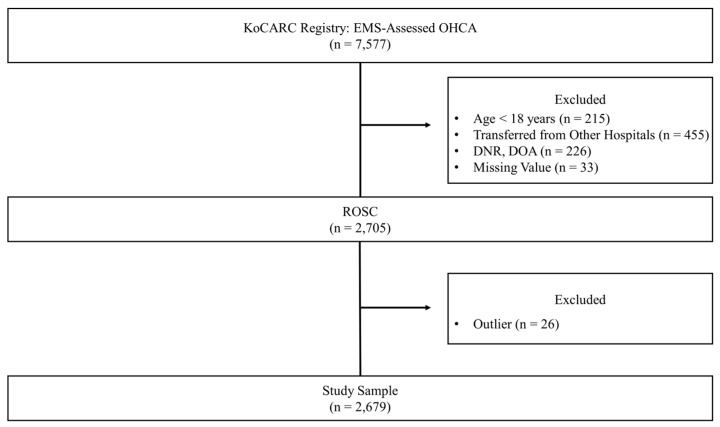
Flow Chart of Study Population.

We conducted a multicenter retrospective observational study using the Korean Cardiac Arrest Research Consortium (KoCARC) registry data for OHCA events from October 2015 to June 2019. The KoCARC is a multicenter collaborative research network in the field of OHCA resuscitation, which started in 2014. The KoCARC registry data are designed to incorporate the variables of existing regional OHCA registries in Utstein templates and are collected via a web-based electronic database system. The KoCARC study population consists of patients visiting its member hospitals and treated by their EMS [15]. The data for this study came from 7577 non-traumatic, EMS-assessed patients who underwent OHCA in the KoCARC registry data with sustained ROSC (Figure 1). Patients with the following characteristics were excluded from this study: an age of 17 or less; a non-medical etiology (e.g., drowning or trauma); a terminal illness; a Do-Not-Resuscitate card previously documented; Death-On-Arrival status; missing information on the neurological outcome; and outliers. The final sample for this study consisted of 2679 patients (Figure 1).

The dependent variable was the binary neurological outcome measured by the cerebral performance category (CPC) score, which EMS personnel assessed and recorded in the registry at discharge: scores 1–2 (good) versus 3–5 (poor) [16]. Based on the clinical relevance in resuscitation practices from the existing literature [2,5,7,8,9,10,11,12,13,14], 68 independent variables were selected across four categories: demographic (age, gender, hypertension, diabetes, family history, etc.); prehospital (witnessed arrest, arrest location, bystander CPR, bystander automated external defibrillator use and defibrillation, first monitored rhythm, prehospital ROSC before emergency room (ER) arrival, etc.); time interval; and in-hospital variables (electrocardiogram, laboratory data, and therapeutic interventions). In-hospital CPR duration was defined as the time between CPR initiation at the hospital and either achieving sustained ROSC or stopping CPR. Our analysis revealed an average missing rate of 13% across the variables. Given this relatively low rate, we chose median imputation for its computational efficiency.

### 2.2. The Korean EMS System

The Korean EMS system is operated by the Korea National Fire Agency. Each ambulance is staffed with two or three EMS providers, including at least one Level-1 Emergency Medical Technician. These technicians can provide CPR with an automated external defibrillator and a limited range of advanced life support, such as endotracheal intubation and epinephrine injection at the scene, under physician medical direction. EMS providers are not legally authorized to declare death or terminate resuscitation attempts in the field unless a patient shows obvious signs of death (e.g., decomposition or rigor mortis).

### 2.3. Statistical Analysis

Machine learning models were selected based on the existing literature [11,12,13,14]. Random forest was chosen as the primary model based on its superior performance compared to logistic regression in terms of accuracy (0.91 vs. 0.89), precision (0.93 vs. 0.91), area under the receiver operating characteristic curve (0.95 vs. 0.91) and sensitivity (0.96 vs. 0.93), as shown in Table 1. Moreover, it is well known for its ability to handle complex, non-linear relationships while providing interpretable variable importance measures. 

The dataset of 2679 cases with complete information was split into training and validation sets with a 75:25 ratio (2009 vs. 670 cases) for final outcomes in Table 1. The validation criteria for the trained models included the following: accuracy (the ratio of correct predictions among 670 cases), precision (the ratio of true positives among predicted positives), the area under the receiver operating characteristic curve (the area under the plot of sensitivity against 1 specificity at various threshold settings) and sensitivity (the ratio of predicted positives among true positives).

The random split and analysis for all participants were repeated 50 times, and the average was used for external validation in Table 1. The training and validation sets with a 75:25 ratio (2009 vs. 670 cases) differed in each run, significantly strengthening the analysis validity when a third test set was unavailable, as in this study. For the decision tree, the impurity criterion was GINI, and the maximum depth was not predetermined. The random forest consisted of 1000 trees, and random forest variable importance (the contribution of an independent variable to the random forest performance) was used to identify major predictors of favorable neurological outcomes. For the artificial neural network, weight optimization was based on the limited-memory Broyden–Fletcher–Goldfarb–Shanno algorithm, and each of two hidden layers had 10 neurons. These hyper-parameters were the default values of the scikit-learn package in Python (https://scikit-learn.org/stable/supervised_learning.html, accessed on 1 December 2024).

**Figure 2:**
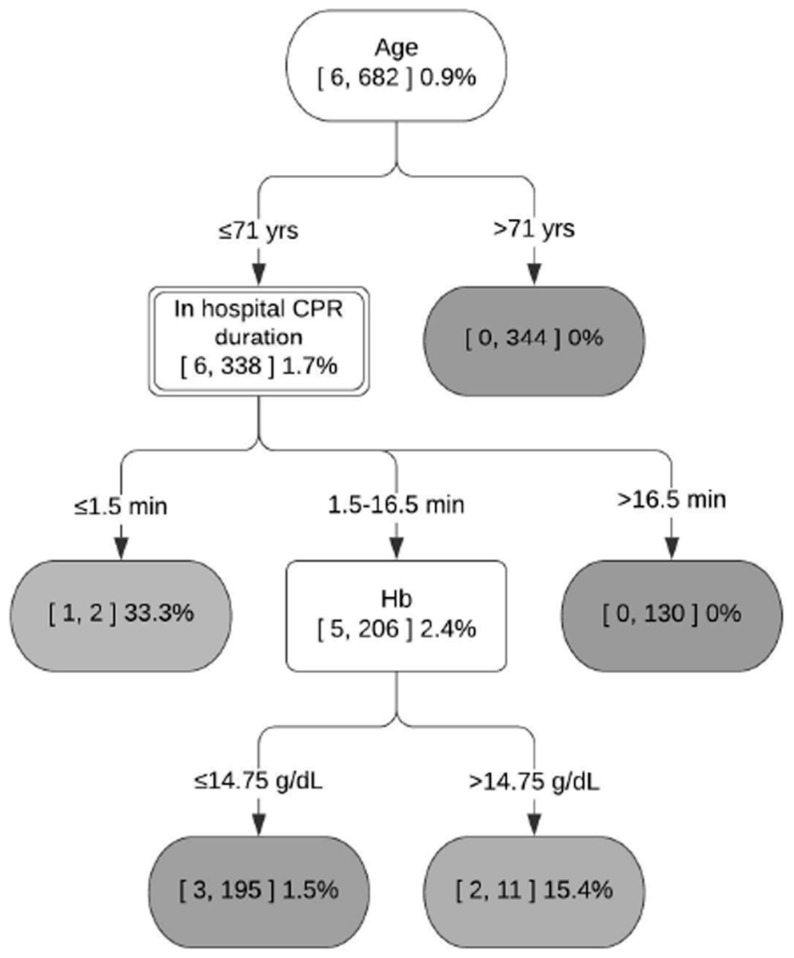
Decision support system for group 3: no prehospital ROSC or the first monitored rhythm of asystole. Terminal nodes are colored with a gradient scale based on the proportion of good outcomes, with darker shades indicating lower proportions of favorable outcomes.

**Figure 3:**
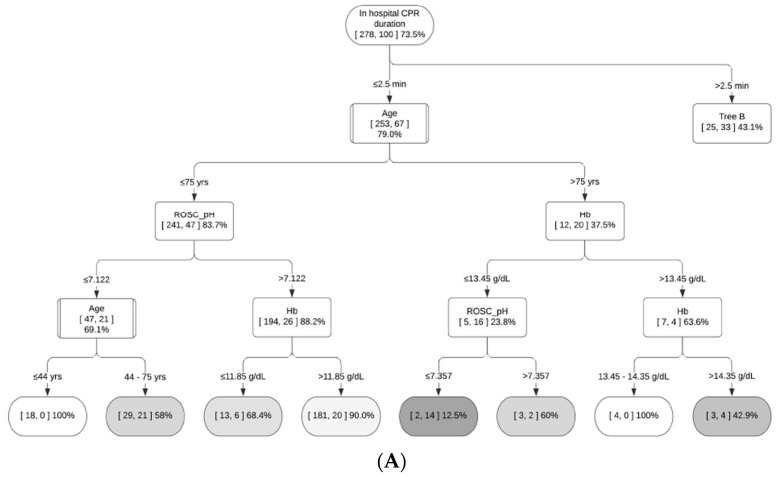

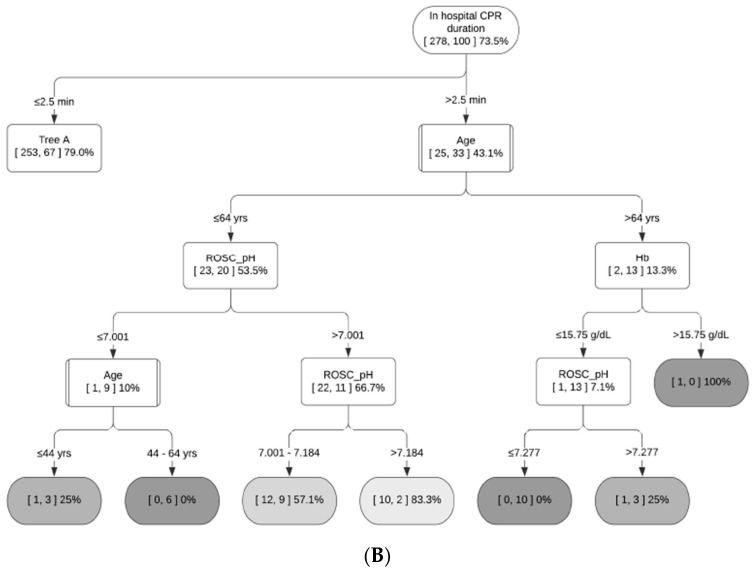
Decision support system for group 4: prehospital ROSC and the first monitored rhythm of VF/pulseless VT. (**A**) In-hospital CPR duration ≤ 2.5 min. (**B**) In-hospital CPR duration > 2.5 min. Terminal nodes are colored with a gradient scale based on the proportion of good outcomes, with darker shades indicating lower proportions of favorable outcomes.

As the final step, the random forest was used to identify major determinants of OHCA outcomes and develop decision support systems for various subgroups (Figure 2 and Figure 3, Figures S1–S4 (Supplementary Figures)). The decision support system figure represents a typical decision tree, consisting of (1) internal nodes (each representing a test on an independent variable), (2) branches (each denoting a test outcome) and (3) terminal nodes (each representing the dependent variable). In each node, the numbers in brackets represent the number of patients with good neurological outcomes (CPC 1–2) and the total number of patients. Terminal nodes are colored with a gradient scale based on the proportion of good outcomes, with lighter shades indicating higher proportions of favorable outcomes (e.g., white representing 100%). This visualization enables the rapid identification of patient subsets with varying prognostic patterns.

R-Studio 1.3.959 (R-Studio Inc.: Boston, MA, USA) and Python 3.5.2 (Centrum voor Wiskunde en Informatica, Amsterdam, The Netherlands) were used for all analyses during 1 March 2020–23 February 2021.

The random split and analysis for all participants were repeated 50 times, and its average was taken for external validation. The training and validation sets with a 75:25 ratio (2009 vs. 670 cases) were different in each run, strengthening the validity of the analysis significantly when a third test set was not available. The impurity criterion was GINI, and the maximum depth was not predetermined for the decision tree. The number of trees was 1000 for the random forest, and random forest variable importance (the contribution of an independent variable for the performance of the random forest) was used for identifying major predictors of favorable neurological outcome. Weight optimization was based on the limited-memory Broyden–Fletcher–Goldfarb–Shanno algorithm, and each of the two hidden layers had 10 neurons in the artificial neural network. These hyper-parameters were the default values of the scikit-learn package in Python (https://scikit-learn.org/stable/supervised_learning.html, accessed on 1 December 2024). R-Studio 1.3.959 (R-Studio Inc.: Boston, MA, USA) and Python 3.5.2 (Centrum voor Wiskunde en Informatica, Amsterdam, The Netherlands) were employed for the analysis during 1 March 2020–23 February 2021.

## 3. Results

Descriptive statistics for the OHCA outcome are shown in Tables S1 and S2 (Supplementary Tables). The median values were 65 years for age, 8 min for in-hospital CPR duration, and 6.94 for post-ROSC pH. Among all of the participants, 19.6% (524) had good neurological outcomes (CPC 1–2), 67.7% (1815) were male, 71.1% (1912) experienced witnessed arrest and 48.5% (1299) received bystander CPR. Additionally, 75.6% (2024) had cardiac arrest rhythm on an in-hospital initial electrocardiogram at ER arrival, 30.2% (810) achieved prehospital ROSC before ER arrival, 27.5% (736) underwent coronary angiography and 29.3% (784) had the first monitored rhythm of VF or pVT (EMS).

Based on random forest variable importance in Table 2, the major determinants of OHCA outcomes were in-hospital CPR duration (0.0824), in-hospital electrocardiogram of cardiac arrest rhythm on ER arrival (0.0692), post-ROSC pH (0.0579), prehospital ROSC before ER arrival (0.0565), coronary angiography (0.0527), age (0.0415), first monitored rhythm (EMS) (0.0402), first monitored rhythm (community) (0.0401), early coronary angiography within 24 h (0.0304) and scene arrival to CPR stop (0.0301). The analysis revealed that the participants could be divided into six subgroups based on prehospital ROSC (before ER arrival) and first monitored rhythm (EMS). A decision tree was developed as a decision support system for each subgroup, incorporating in-hospital CPR duration, post-ROSC pH, age and hemoglobin (Figure 2 and Figure 3, Figures S1–S4 (Supplementary Figures), Table S4, a Supplementary Table).

For example, Figure 2 presents a decision support system for participants without prehospital ROSC and with the first monitored rhythm of asystole. Age comes first in this figure because it provides the most effective data separation. The splitting criterion of 71 years was selected as it provided better data separation than alternative criteria such as 75 or 64 years. This subgroup shows the lowest proportion of patients with CPC 1–2 (1.7%) among the six subgroups. But the proportion of patients with CPC 1–2 can be as high as 33.3% in this subgroup: with the sequence of the age of 71 years or less, then the in-hospital CPR duration is 1.5 min or less. The effective cut-off points for this subgroup are 71 years for age and 16.5 min for in-hospital CPR duration. On the contrary, Figure 3 is a decision support system for patients with prehospital ROSC and the first monitored rhythm of VF or pVT. This subgroup has the highest proportion of patients with CPC 1–2 (70.9%) among the six subgroups. However, the proportion of patients with CPC 1–2 can be as low as 0% in this subgroup, with the sequence of in-hospital CPR duration longer than 2.5 min, age older than 64 years and hemoglobin of 15.75 g/dL or less; then, the post-ROSC pH is 7.277 or less. The effective cut-off points for this subgroup are 2.5 min for in-hospital CPR duration, 64/75 years for age, 15.75 g/dL for hemoglobin and 7.184/7.277 for post-ROSC pH.

## 4. Discussion

The survival and neurological outcomes of OHCA patients showed a steady long-term improvement with intensive effort for the enhancement of the Chain of Survival and the provision of guideline-centered care from community to hospital [6,17,18]. The survival rate of OHCA patient is estimated to have increased from 5.7% to 8.3% during 2005–2012 in the United States [17]. Similarly, the rate of survival (or a good neurological outcome) is reported to have risen from 2.3% to 8.7% (or from 0.6% to 5.4%) in Korea [6]. However, this trend recently slowed down despite recent advances in post-resuscitation care, and personalized approaches to in-hospital care would be considered as one way to make further improvements.

The existing literature based on conventional statistical models and scoring systems reports that the main predictors of good neurological outcomes in OHCA patients are younger age, male status, being witnessed by a bystander or EMS provider, the provision of bystander CPR, the first monitored rhythm of VF or pVT and prehospital ROSC [5,7,8,9,10]. These results are consistent with those of this study in general, given that age, first monitored rhythm and prehospital ROSC ranked within the top 10 in terms of random forest variable importance in this study. However, most of these classical predictors from conventional models are determined in the prehospital phase. Moreover, these prehospital predictors do not provide detailed information about decisions regarding invasive intervention in an acute period after achieving ROSC [5,9,10,19,20]. While machine learning in recent studies outperformed the traditional approaches, they primarily relied on the same classical variables in the prehospital phase [11,12,14,21,22], limiting their utility for acute-phase decision making. This study endeavors to overcome this critical weakness of the existing literature by putting more focus on in-hospital variables, including time factors and laboratory results. Our results in OHCA patients with ROSC showed the importance of in-hospital variables, in the order of in-hospital CPR duration, post-ROSC pH, prehospital ROSC, coronary angiography (CAG), age and first monitored rhythm.

Specifically, this study followed previous studies to highlight four biological variables associated with good outcomes; that is, in-hospital CPR duration, post-ROSC pH, age and hemoglobin. Several studies employed various biological variables during 3–5 post-arrest days for neurological prognostication, i.e., arterial pH, hemoglobin, serum lactate, biomarkers, brain computed tomography and the Bispectral Index [8,23,24,25,26]. Likewise, a previous study demonstrated the importance of age, adrenaline dose, serum creatinine level and time to ROSC during the first three days for predicting outcomes in successfully resuscitated patients [26]. Machine learning models based on these biological predictors, in addition to pre-determined factors, can aid in evaluating the risk–benefit of interventions such as extracorporeal CPR or withdrawal of life-sustaining treatments. Moreover, Al-Dury et al. showed that a subgroup analysis based on the first monitored rhythm in the prehospital stage would contribute to better prediction outcomes and different importance results in OHCA patients: the top five predictors in the case of the shockable rhythm were time to defibrillation, age, defibrillation, arrest location and time from arrest to CPR; the five most important variables in the case of the non-shockable rhythm were age, defibrillation before EMS arrival, time to EMS arrival, arrest location and the cause of arrest [27].

This study demonstrates the utility of biological variables in outcome prediction, and it can be noted that incorporating additional in-hospital parameters would bring more improvement in model accuracy and clinical effectiveness. Recent evidence highlights the critical role of oxygenation status in cardiac arrest outcomes [28,29]. A comprehensive meta-analysis demonstrated that severe post-arrest hyperoxemia during the first 36 h post-arrest significantly impacts both mortality (OR 1.32 [95% CI 1.11,1.57]) and neurological outcomes (OR 1.37 [95% CI 1.01,1.86]) [28]. In a similar vein, the TTM2 trial analysis revealed more precise thresholds for both hypoxemia (69 mmHg) and hyperoxemia (195 mmHg) that influence survival [29]. These findings suggest that predictive models should incorporate not only discrete oxygen measurements but also temporal exposure patterns and the cumulative oxygen burden, potentially improving the precision of outcome prediction in post-cardiac-arrest care.

Existing machine learning research focused on the superior performance of machine learning compared to logistic regression, and it was also found in this study that the random forest performs better than does logistic regression in terms of accuracy and the area under the receiver operating characteristic curve (i.e., 0.91, 0.96 vs. 0.89, 0.92). However, this study went beyond the scope of conventional studies by using machine learning to form various subgroups as the first stage and developing a decision support system for each subgroup as the second stage: (1) the total patients were divided into six subgroups in terms of prehospital ROSC and first monitored rhythm by EMS, given that these two variables were reported to be the key predictors of neurological outcomes in the existing literature [5,7,8,9,10] and this study alike; (2) then, a decision tree from a random forest was developed as a decision support system for each subgroup to find its own effective cut-off points regarding continuous variables, such as in-hospital CPR duration, post-ROSC pH, age and hemoglobin, from the top-ranked variables. For instance, it was found in this study that a subgroup with no prehospital ROSC and the first monitored rhythm of asystole has the lowest proportion of patients with CPC 1–2 (1.3%) among the six subgroups. Based on the results of this study, however, the proportion of patients with CPC 1–2 can be as high as 33.3% in this subgroup: with the sequence of the age of 71 years or less, then the in-hospital CPR duration is 1.5 min or less. The effective cut-off points for this subgroup are 71 years for age and 16.5 min for in-hospital CPR duration. On the contrary, it was found that a subgroup with prehospital ROSC and the first monitored rhythm of VF or pVT has the highest proportion of patients with CPC 1–2 (70.9%) among the six subgroups. This finding is consistent with those of the existing literature [2,7,9,10]. However, the proportion of patients with CPC 1–2 can be as low as 0% in this subgroup, with the sequence of in-hospital CPR duration longer than 2.5 min, age older than 64 years and hemoglobin of 15.75 g/dL or less; then, the post-ROSC pH is 7.277 or less. The effective cut-off points for this subgroup are 2.5 min for in-hospital CPR duration, 64/75 years for age, 15.75 g/dL for hemoglobin and 7.184/7.277 for post-ROSC pH.

This study advances OHCA prognostication through subgroup-specific decision support systems that enable precise risk stratification. Our analysis revealed distinct prognostic patterns within traditionally defined risk groups. In high-risk populations (no prehospital ROSC or first monitored rhythm of asystole), specific variable combinations (age ≤ 71 years and in-hospital CPR duration ≤ 1.5 min) identified a subset with 33.3% favorable outcomes despite an overall group favorable outcome rate of 1.3%. Similarly, in conventionally low-risk groups (prehospital ROSC and first monitored rhythm of VF or pVT), specific variable combinations identified subsets with poor prognosis, guiding appropriate intervention timing. These findings highlight the ability to derive optimal cut-off points for continuous variables (e.g., in-hospital CPR duration, post-ROSC pH, age and hemoglobin) in each subgroup, enabling personalized treatment strategies. Notably, this study demonstrated that the ranking and importance of predictors varied across subgroups, advocating for a more refined, individualized treatment approach instead of a one-size-fits-all model. Unlike traditional logistic regression, which assumes ceteris paribus conditions where variables remain constant during analysis, decision trees and random forests capture sequential information critical for both statistical analysis and clinical application. To our best knowledge, considering sequential information of trees and associations among key variables in each of the six subgroups represents an optimal approach in its own right. This methodology reveals the importance of variables within complicated heterogenous parameters in clinical settings and would be helpful to optimize the efficiency of care through robust subgroup-based prognostication. However, the clinical implementation of these systems may face challenges, including the integration of machine learning models into existing workflows, the need for clinician training, and the variability of available resources across institutions. Future studies should focus on validating these decision support systems in real-world settings and developing implementation frameworks to ensure their seamless integration into clinical practice.

While these decision support systems aim to improve prognostication and guide clinical interventions in OHCA patients, ethical considerations are paramount, particularly regarding the withdrawal of life-sustaining treatments. The stratification of patients should serve as an adjunct to clinical judgment rather than a deterministic tool, emphasizing shared decision making among healthcare teams, patients (when possible) and families. Implementation must consider cultural and institutional variations in end-of-life care practices to ensure equitable treatment decisions aligned with principles of patient autonomy, beneficence and justice.

This study still has some limitations. Firstly, this study did not consider the etiologies of the OHCA outcome. Secondly, this study included OHCA outcome as a binary variable, and considering the multinomial OHCA outcome would bring more profound clinical insights. Thirdly, more validation for additional data would improve the external validity of this study, given that machine learning is a data-driven approach. Fourthly, OHCA outcome can vary across medical institutions with different levels and volumes [30]. The KoCARC registry data, drawn exclusively from general hospitals, restricts the generalizability of results across all medical institutions and introduces potential selection bias through the exclusion of certain patient groups. Fifthly, the performance of the random forest was not as good for the subgroups as for all participants, given that the size of each subgroup was much smaller than that of all participants. The median accuracy, precision and area under the receiver operating characteristic curve for the six subgroups were 0.86, 0.94 and 0.60, respectively (Table S3). Collecting more data for each subgroup is expected to improve the model performance and reliability. Furthermore, it is important to consider the trade-offs inherent in using machine learning models. While random forest provides improved prediction accuracy and allows for subgroup-specific insights, this comes at the cost of greater complexity compared to traditional methods. Balancing these trade-offs to optimize both predictive power and clinical interpretability remains a key area for future research. Sixthly, the results of this study indicate that effective cut-off points for good neurological outcomes regarding in-hospital CPR duration, post-ROSC pH, age and hemoglobin are likely to be different across subgroups (every subgroup has its own sequential information and associations among key variables). It will be an important topic for future research to develop more effective ways of clinical interpretation and implication for each subgroup. Seventhly, it can be noted that the average rate of missing values in the 68 independent variables was 13%, and these missing values were replaced by their medians in this study. While median imputation was chosen for its computational efficiency, there are other approaches of imputation, and comparing their effects on model performance is expected to further the boundary of knowledge on this topic. Eighthly, extending this study to include boosting and deep learning would be a significant contribution to this line of research.

## 5. Conclusions

We identified the major determinants of favorable neurological outcomes in successfully resuscitated OHCA patients using machine learning. This study demonstrated the strengths of the random forest as an effective decision support system for each stratified subgroup (prehospital ROSC and first monitored rhythm by EMS) to find its own optimal cut-off points for major in-hospital variables (in-hospital CPR duration, post-ROSC pH, age and hemoglobin).

## Figures and Tables

**Table 1:** Model performance for the prediction of OHCA outcome in 50 runs.

Model	Accuracy	Precision	AUC	AUC-L	AUC-U	Sensitivity
Logistic Regression	0.8888	0.91	0.91	0.91	0.91	0.93
Decision Tree	0.8624	0.90	0.79	0.78	0.80	0.91
Naive Bayes	0.8395	0.96	0.89	0.89	0.90	0.84
Random Forest-1000 Trees	0.9115	0.93	0.95	0.94	0.96	0.96
Support Vector Machine	0.8063	0.86	0.88	0.87	0.88	1.00
Artificial Neural Network	0.8559	0.89	0.87	0.86	0.87	0.94

Abbreviations: AUC, area under the receiver operating characteristic curve. AUC-L/U, AUC lower/upper bound for the 95% confidence interval.

**Table 2:** Random forest and logistic regression results.

	Random Forest			Logistic Regression
Variables	VI-Value	Rank	Exp (Coef)	Z
In-hospital CPR Duration	0.0824	1	0.9970	0.1845
In-hospital Electrocardiogram on ER Arrival	0.0692	2	0.5387	1.7338
Post-ROSC pH	0.0579	3	1.0005	1.8176
Prehospital ROSC before ER Arrival	0.0565	4	7.1254	* 4.452
CAG	0.0527	5	0.2711	* −4.0550
Age	0.0415	6	0.9629	* −5.2174
First Monitored Rhythm (EMS)	0.0402	7	0.8686	−0.1884
First Monitored Rhythm (Community)	0.0401	8	0.1613	* −2.3036
Early CAG (within 24 h)	0.0304	9	0.5334	* −2.1087
Scene Arrival to CPR stop (min)	0.0301	10	0.9903	−0.5712
Initial pH	0.0291	11	1.5732	1.1674
aPTT	0.0280	12	1.0007	0.1496
Hb	0.0227	13	1.0288	1.2873
Epinephrine Dose, Total	0.0226	14	0.9230	* −2.1357
Witness to ER Arrival (min)	0.0195	15	1.0009	0.3596
Post-ROSC Lactate	0.0190	16	1.0005	0.1044
Potassium	0.0185	17	1.0032	0.1871
Down Time	0.0184	18	0.9845	−1.8926
Scene Arrival to ER Arrival (min)	0.0174	19	1.0026	0.1430
PLT	0.0154	20	1.0019	1.2267

Abbreviations/notes: * *p* < 0.05; VI, variable importance; Exp (Coef), exponential value of the estimated coefficient; CPR, cardiopulmonary resuscitation; ER, emergency room; ROSC, return of spontaneous circulation; CAG, coronary angiography; EMS, emergency medical services.

## Data Availability

The datasets used and/or analyzed during the current study are available from the corresponding author on reasonable request.

## Supplementary Materials

The following supporting information can be downloaded at https://www.mdpi.com/article/10.3390/jcm13247600/s1, Table S1. Descriptive statistics on categorical variables (total 2679, N (%)); Table S2. Descriptive statistics on continuous variables; Table S3. Accuracy, precision and AUC in each subgroup; Table S4. Descriptive statistics in each subgroup; Figure S1. Decision support system for group 1: no prehospital ROSC or the first monitored rhythm of VF/pulseless VT; Figure S2. Decision support system for group 2: no prehospital ROSC or the first monitored rhythm of PEA; Figure S3. Decision support system for group 5: prehospital ROSC and the first monitored rhythm of PEA; Figure S4. Decision support system for group 6: prehospital ROSC and the first monitored rhythm of asystole.

## Abbreviations

CAG	Coronary Angiography
CPC	Cerebral Performance Category
CPR	Cardiopulmonary Resuscitation
ED	Emergency Department
EMS	Emergency Medical Services
ER	Emergency Room
KoCARC	Korean Cardiac Arrest Research Consortium
KACPR	Korean Association of Cardiopulmonary Resuscitation
OHCA	Out-of-Hospital Cardiac Arrest
ROSC	Return Of Spontaneous Circulation
VF	Ventricular Fibrillation
pVT	Pulseless Ventricular Tachycardia
