# Supplementary material for: A Machine Learning-Based Decision Support System for the Prognostication of Neurological Outcomes in Successfully Resuscitated Out-of-Hospital Cardiac Arrest Patients
Source: J Clin Med. 2024 Dec 13;13(24):7600. doi: 10.3390/jcm13247600 (PMC11676625; doi:10.3390/jcm13247600)

Supplementary Table S1. Descriptive Statistics on Categorical Variables (Total 2679, N (%))

Variable	Yes	No
Male	1815 (67.7)	864 (32.3)
Hypertension	1142 (42.6)	1537 (57.4)
Diabetes Mellitus	721 (26.9)	1958 (73.1)
Dyslipidemia	163 (6.1)	2516 (93.9)
Family History of Cardiac Arrest		1407 (52.5)
Parent	20 (0.7)	
Sibling	14 (0.5)	
Unknown	1238 (46.2)	
Family History of ACS		1372 (51.2)
Parent	41 (1.5)	
Sibling	20 (0.7)	
Unknown	1246 (46.6)	
Family History of CVA		1375 (51.4)
Parent	54 (2)	
Sibling	11 (0.4)	
Unknown	1239 (46.2)	
Family History of Arrhythmia		1406 (52.5)
Parent	8 (0.3)	
Sibling	3 (0.1)	
Unknown	1262 (47.1)	
Witnessed Arrest	1912 (71.4)	767 (28.6)
Arrest Location		
Home/Residence	1453 (54.2)	
Street/Highway	317 (11.8)	
Public Building	143 (5.3)	

Industrial/Workplace	143 (5.3)	
Sports/Recreation Event	122 (4.6)	
Other	96 (3.6)	
Unknown	417 (15.6)	
Bystander CPR	1299 (48.5)	1380 (51.5)
Bystander AED	44 (1.6)	2635 (98.4)
First Monitored Rhythm (Community)		
VF/Pulseless VT	762 (28.4)	
PEA	658 (24.6)	
Asystole	1017 (38)	
Unknown	242 (9)	
First Monitored Rhythm (EMS)		
VF/Pulseless VT	784 (29.3)	
PEA	1034 (38.6)	
Asystole	675 (25.2)	
Unknown	186 (6.9)	
Pre-hospital ROSC before ER Arrival	810 (30.2)	1869 (69.8)
ROSC by EMS Provider	512 (19.1)	2167 (80.9)
Defibrillation by EMS Provider	677 (25.3)	2002 (74.7)
Drug Use by EMS Provider	318 (11.9)	2311 (86.3)
Unknown	50 (1.9)	
Epinephrine Use by EMS Provider	314 (11.7)	2365 (88.3)
Airway Management by EMS Provider	2100 (78.4)	579 (21.6)
Intubation by EMS Provider	195 (7.3)	2484 (92.7)
Supraglottic Airway by EMS Provider	1243 (46.4)	1436 (53.6)
Mechanical CPR	299 (11.2)	2380 (88.8)
In-hospital Electrocardiogram on Arrival at ER		

Non-Arrest Rhythm after ROSC	655 (24.4)	
Cardiac Arrest Rhythm	2024 (75.6)	
ECMO Apply	123 (4.6)	2546 (95)
Transfer after ECMO Apply	10 (0.4)	
ECMO Activation Success	55 (2.1)	867 (32.4)
Unknown	1757 (65.5)	
Reperfusion Attempted	554 (20.7)	2125 (79.3)
CAG	736 (27.5)	1943 (72.5)
Early CAG (within 24 hours)	659 (24.6)	2020 (75.4)
Inotropics Use after ROSC	1763 (65.8)	916 (34.2)
ECMO Apply after ROSC	81 (3)	2598 (97)
TTM after ROSC	525 (19.6)	2154 (80.4)
Thrombolytic Agent after ROSC	233 (8.7)	2446 (91.3)
PCI after ROSC	269 (10)	2410 (90)
ICD after ROSC	61 (2.3)	2618 (97.7)
TCP	21 (0.8)	2658 (99.2)
Pacemaker	46 (1.7)	2633 (98.3)
Intra-Aortic Balloon Pump	11 (0.4)	2668 (99.6)
CABG	33 (1.2)	2646 (98.8)
CPC 1-2	524 (19.6)	2155 (80.4)

Abbreviations: ACS, acute coronary syndrome; AED, automated external defibrillator; CABG, coronary artery bypass graft; CAG, coronary angiography; CPC, cerebral performance category; CPR, cardiopulmonary resuscitation; CVA, cerebrovascular accident; ECMO, extracorporeal membrane oxygenation; EMS, emergency medical services; ICD, intracardiac defibrillator; PCI, percutaneous coronary intervention; PEA, pulseless electrical activity; ROSC, return of spontaneous circulation; TCP, transcutaneous pacing; TTM, targeted temperature management; VF, ventricular fibrillation; VT, ventricular tachycardia.

Supplementary Table S2. Descriptive Statistics on Continuous Variables

Variables	Mean	Median (IQR)
Age	64.48 ± 15.4	65 (54-76)
Witness to ER Arrival (min)	36.91 ± 67.68	30 (23-37)
Call to Scene (min)	7.9 ± 4.53	7 (5-9)
Scene Arrival to Scene Departure (min)	11.97 ± 5.44	12 (10-12)
Scene Departure to ER Arrival (min)	10.15 ± 5.56	9 (8-11)
Scene Arrival to CPR Stop (min)	37.61 ± 21.95	32 (24-43)
In-hospital CPR Duration	14.79 ± 19.01	8 (2-18)
ER Arrival to Sustained ROSC (min)	14.48 ± 15.97	11 (9-12)
Down Time	55.82 ± 62.62	54 (37-54)
Epinephrine Dose until Sustained ROSC	2.56 ± 3.13	1 (1-3)
Epinephrine Dose, Total	4.82 ± 4.48	4 (2-6)
Hb	12.05 ± 2.28	12.1 (11.6-12.9)
PLT	184.47 ± 64.85	173 (173-198)
aPTT	52.85 ± 27.66	49.6 (41.4-49.6)
CK-MB	8.99 ± 31.05	4 (2.15-5.62)
Glucose	264.86 ± 112.54	250 (239-275)
K	5.16 ± 1.27	5.4 (4.3-5.4)
Calcium	8.59 ± 2.33	8.6 (8.5-8.6)
Initial pH	6.99 ±0.167	6.94 (6.94-7.05)
Post-ROSC pH	7.037 ± 0.19	7.022 (6.94-7.14)
Post-ROSC Lactate	13.86 ± 19.53	11 (9.1-12.6)

Abbreviations: CPR, cardiopulmonary resuscitation; ER, emergency room; ROSC, return of spontaneous circulation

Supplementary Table S3. Accuracy, Precision and AUC in Each Subgroup

Subgroup	Accuracy	Precision	AUC
1	0.8857	0.98	0.88
2	0.9275	0.93	0.50
3	0.9696	0.98	0.51
4	0.6693	0.60	0.62
5	0.5938	0.75	0.57
6	0.8276	0.96	0.72
Median	0.8567	0.94	0.60

Abbreviations: AUC Area Under the Receiver-Operating-Characteristic Curve

Subgroup 1, 2, 3 No Prehospital ROSC, First Monitored Rhythm of VF/PVT, PEA, Asystole

Subgroup 4, 5, 6 Prehospital ROSC, First Monitored Rhythm of VF/PVT, PEA, Asystole

Supplementary Table S4. Descriptive Statistics in Each Sub-Group

Variables	CPC 1-2 (%)	In hospital CPR duration (average min)	post-ROSC pH (average)	Age (average years)	Hb (average g/dL)
1	11.8	23	7.02	61	12.41
2	5.1	18	7.00	67	11.86
3	1.3	19	6.97	69	11.36
4	70.9	4	7.20	56	13.31
5	24.6	6	7.07	64	12.26
6	13.2	14	6.98	64	11.54

Abbreviations: CPC, cerebral performance category; CPR, cardiopulmonary resuscitation; ROSC, return of spontaneous circulation; Hb, hemoglobin

Figure S1. Decision Support System for Group 1 - No Prehospital ROSC and the First Monitored Rhythm of VF/Pulseless VT

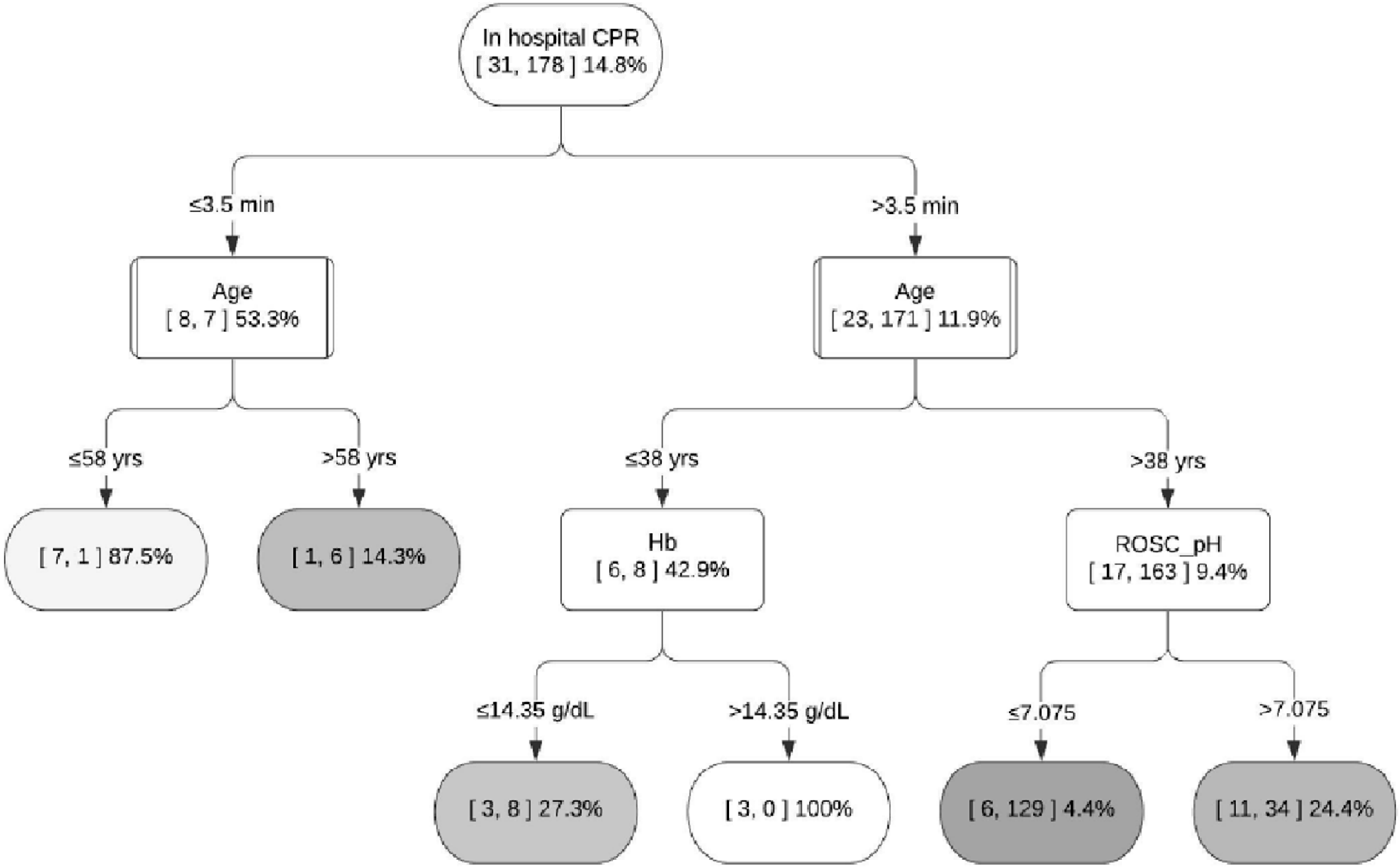

Figure S2. Decision Support System for Group 2 - No Prehospital ROSC and the First Monitored Rhythm of PEA

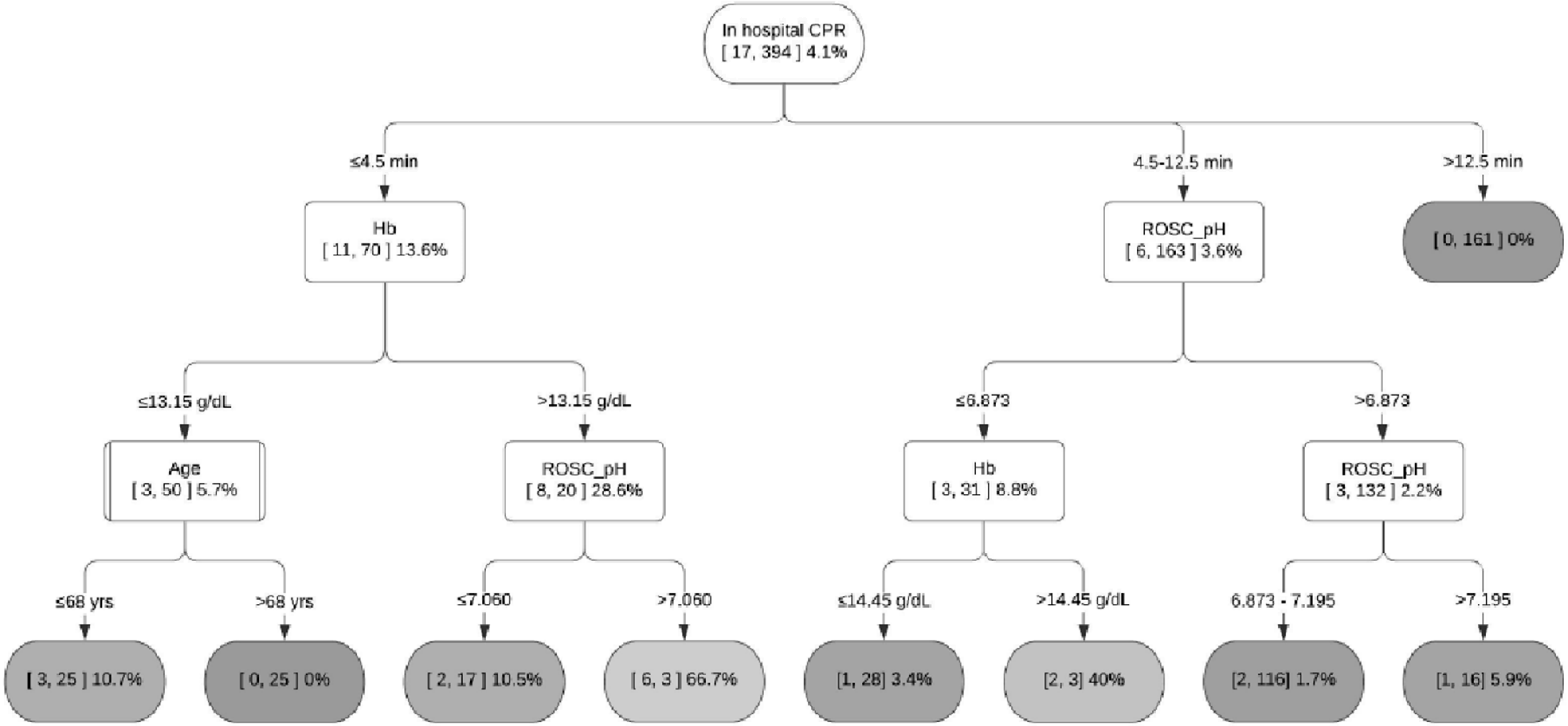

Figure S3. Decision Support System for Group 5 - Prehospital ROSC and the First Monitored Rhythm of PEA

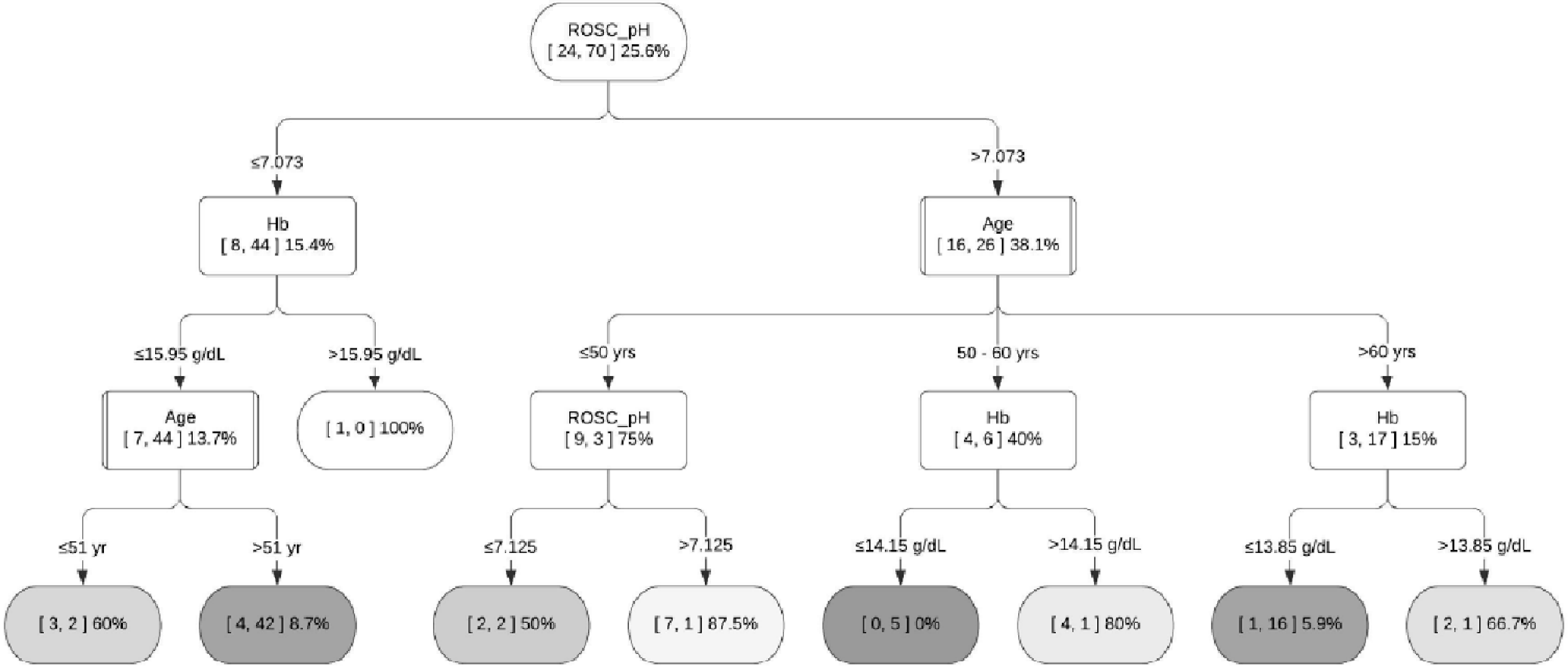

Figure S4. Decision Support System for Group 6 - Prehospital ROSC and the First Monitored Rhythm of Asystole

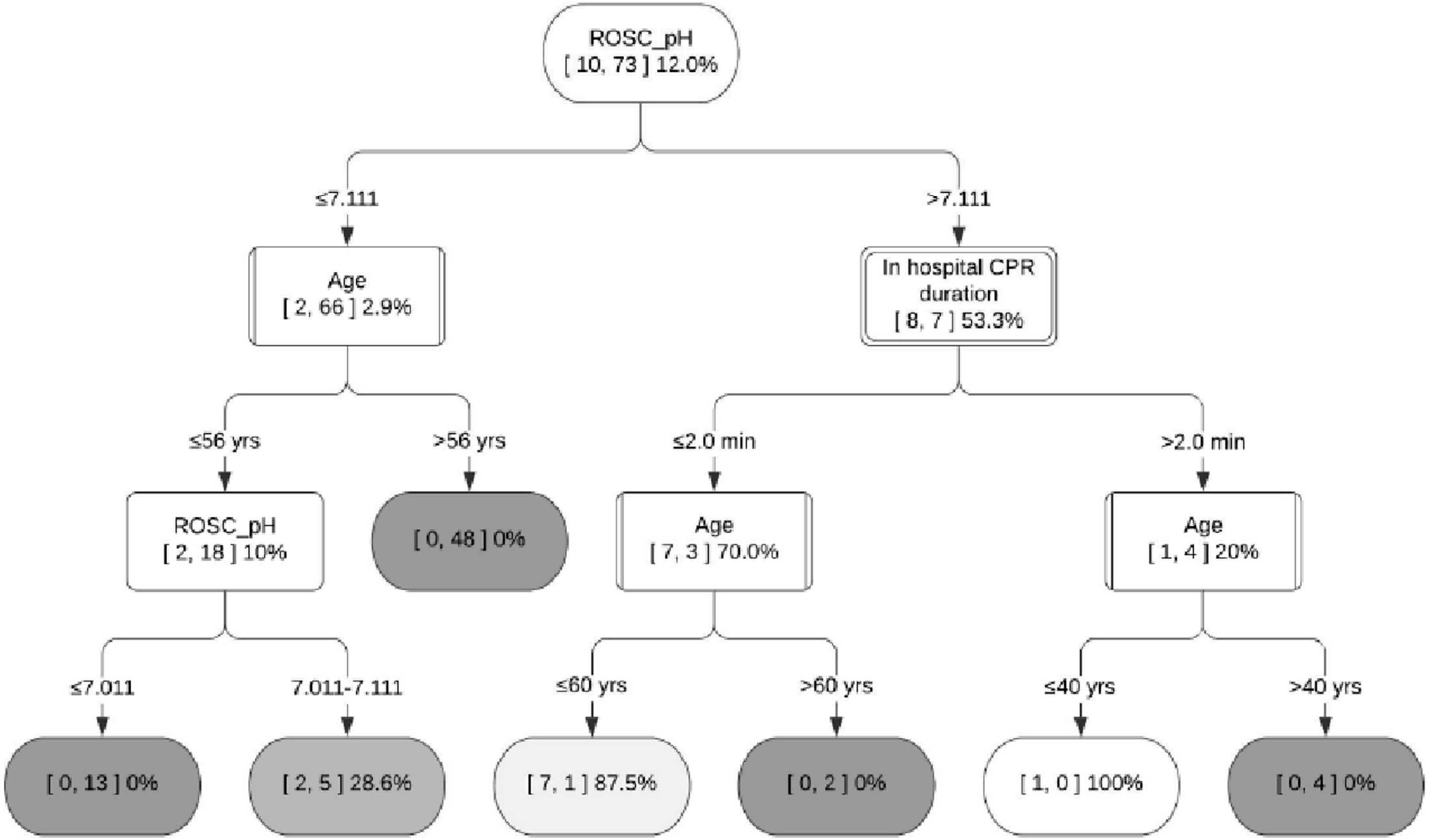

Supplement: Supplementary file 1 [file jcm-13-07600-s001.zip › jcm-3315858-supplementary.pdf]
